# Heart Valve Surgery in Antiphospholipid Syndrome Patients—Morbidity and Mortality

**DOI:** 10.3390/life13040891

**Published:** 2023-03-27

**Authors:** Tali Eviatar, Stanley Niznik, Ori Elkayam, Yanai Ben-Gal, Ronen Shavit, Ehud Raanani, Nancy Agmon-Levin, Daphna Paran

**Affiliations:** 1 Rheumatology Department, Tel-Aviv Sourasky Medical Center, Tel Aviv 6423906, Israel; 2 Sackler Faculty of Medicine, Tel Aviv University, Tel Aviv 6997801, Israel; 3 The Zabludowicz Center for Autoimmune Diseases, Sheba Medical Center, Tel Hashomer, Ramat-Gan 52621, Israel; 4 Department of Cardiothoracic Surgery, Tel-Aviv Sourasky Medical Center, Tel Aviv 6423906, Israel; 5 Department of Cardiac Surgery, Leviev Cardiothoracic and Vascular Center, Sheba Medical Center, Tel Hashomer, Ramat-Gan 52621, Israel

**Keywords:** antiphospholipid syndrome, systemic lupus erythematosus, mitral valve replacement, valve surgery

## Abstract

Objectives: To assess valve surgery outcomes in antiphospholipid syndrome (APS). Methods: A retrospective study assessing complications and mortality rate and possible factors associated with adverse outcomes of APS patients undergoing valve surgery in two tertiary medical centers. Results: Twenty-six APS patients (median age at surgery 47.5 years) who underwent valve surgery were detected, of whom 11 (42.3%) had secondary APS. The mitral valve was most commonly involved (*n* = 15, 57.7%). A valve replacement was performed in 24 operations (92.3%), 16 of which (66.7%) were mechanical valves. Fourteen (53.8%) patients sustained severe complications, and four of them died. The presence of mitral regurgitation (MR) was associated with severe complications and mortality (odds ratio (95% confidence interval) 12.5 (1.85–84.442), *p* = 0.008, for complications. All deceased patients had MR (*p* = 0.033). The presence of Libman-Sacks endocarditis (LSE) (7.333 (1.272–42.294), *p* = 0.045), low C3 (6.667 (1.047–42.431), *p* = 0.05) and higher perioperative prednisone doses (15 ± 21.89 vs. 1.36 ± 3.23 mg/day, *p* = 0.046) were also associated with complications. A lower glomerular filtration rate (GFR) was associated with mortality (30.75 ± 19.47 vs. 70.68 ± 34.44 mL/min, *p* = 0.038). Conclusions: Significant morbidity and mortality were observed among APS patients undergoing valve surgery. MR was associated with mortality and complications. LSE, low complement and higher doses of corticosteroids were associated with complications, while a low GFR was associated with mortality.

## 1. Introduction

Antiphospholipid syndrome (APS) is an acquired autoimmune, hypercoagulability syndrome characterized by thrombotic and/or obstetric manifestations in association with persistent antiphospholipid autoantibodies (aPL, i.e., anticardiolipin [aCL], anti-β-2glycoprotein I [aB2GPI], lupus anticoagulant [LA]) [1], with an estimated prevalence of 40–50 patients per 100,000 persons [1,2]. Cardiac involvement is prevalent in primary APS or APS secondary to systemic lupus erythematosus (SLE), ranging from 30% to 80%, depending upon the definition and diagnostic methods used [3,4,5]. Cardiac disease in APS includes criteria manifestations, such as myocardial infarction (MI) and intra-cardiac thrombus, as well as “non-criteria” manifestations [6,7], such as myocardial dysfunction, pulmonary hypertension and valvular involvement, that when estimated by transthoracic echocardiography can be found in 33–50% of APS patients [3,4,5,8,9,10,11]. In the course of 5 years, new cardiac involvement was detected by transesophageal echocardiography in 25–36% of primary APS patients [12,13], while in patients with SLE and/or APS, new valvopathy was found in 26.8% after 10 years of follow-up [8]. Cardiac and valvular manifestations are associated with significant morbidity, including thromboembolic phenomena and increased mortality [3,11,14]. Heart valve disease may present with valvular leaflet thickening, stenosis, or regurgitation and Libman-Sacks endocarditis (LSE) [8,9,10,15], with an estimated prevalence in SLE of around 10%, that may progress to severe disease necessitating valve surgery in 4–6% of the patients [4,10,16,17,18]. Surgical-related thrombotic and/or hemorrhagic complications have been described in patients with primary or secondary APS [19,20], however, data regarding valve surgery outcomes in APS are still lacking and rely mostly upon case series and case reports [16,21,22].

This descriptive retrospective study aimed to assess the outcome and prognosis of cardiac valve surgery in patients with primary or secondary APS in two tertiary medical centers in Israel, and to identify factors that may be associated with adverse outcomes.

## 2. Patients and Methods

### 2.1. Patients

All adult patients, aged 18 years or older at the time of surgery, with a diagnosis of APS, either primary or secondary to SLE, who underwent valve surgery at the Tel Aviv Sourasky Medical Center (TLVMC) or Sheba Tel-Hashomer Medical Center, Israel since 1992 were included in this retrospective analysis. Patients were required to fulfill the Sapporo criteria [1], or to lack any other more reasonable or suitable etiology for their valve disease. We included patients with a post-surgical follow-up of at least 30 days, as well as patients who had died within this time period.

### 2.2. Methods

The primary objective was to assess the rate of severe complications and mortality. An exploratory objective was to identify the factors associated with morbidity and mortality in our cohort.

We collected demographic and clinical data before surgery and data regarding the outcome following surgery. The retrieved information included:

1. Demographic and clinical data for each patient were collected from electronic case files, including APS manifestations, the aPL profile, other immunologic serologies (antinuclear antibodies, anti-double stranded DNA antibodies, complement levels) and periprocedural blood test results up to 60 days before surgery (blood count indices, creatinine, direct Coombs test). The presence of aCL and aB2GPI of the IgG and IgM isotypes were measured by enzyme-linked immunosorbent assay (ELISA) or by a multiplex system. All kits were commercial (ELISA—aB2GPI by AESKU Diagnosis, and aCL by Varelisa; Bioplex both aB2GPI and aCL by BIORAD). The results were considered positive if they were above the upper limit of normal (ULN) as specified for each kit according to the manufacturer’s instructions (IgG phospholipid units or IgM phospholipid units), or >99th percentile in a minimum of two tests performed at least 12 weeks apart. They were considered clinically significantly positive if above 40 MPL or GPL (which is 2–4 fold above the ULN), as specified by the Sapporo criteria [1]. LA activity was detected by coagulation assays in routine use at each center, and it was consistent with the International Society of Thrombosis and Hemostasis guidelines [23]. LA activity was measured by LA-responsive activated partial thromboplastin time (aPTT) aPL (by Stago, with confirms by Actin FS kit by Siemens) up to 2016, and by Silica Clotting time and dilute Russel Viper Venom time (kit by Werfen) after 2016. In the case of anticoagulation treatment or spontaneous INR > 1.5, the patient’s plasma was mixed with normal plasma in order to reduce false positivity. aPL positivity was defined as single, double, or triple positive according to the number of different positive tests obtained.
2. Cardiac characteristics: LSE based upon the local echocardiographic report [24], coronary pathologies based upon coronary angiography findings and indications for surgery.
3. Medical treatment before and after surgery: mode of anticoagulation (low molecular weight heparin [LMWH], vitamin K antagonists [VKA], direct oral anticoagulants [DOAC], aspirin, glucocorticoids [intravenous pulse therapy, oral prednisone or prednisone equivalent dosage]) and immunosuppressants.
4. The valve replaced or repaired and the type of valve inserted (mechanical or biologic).
5. New York Heart Association functional classification (NYHA) was reported for patients before surgery.
6. Probability of pulmonary hypertension (PHTN) as assessed by echocardiography was graded as none when systolic pulmonary artery pressure estimation was below 30 mmHg, low if 30–39 mmHg, intermediate if 40–59 mmHg and high if above 60 mmHg [25].
7. Adverse outcomes following surgery were defined as early (if they occurred up to 30 days post-surgery) or late (if they occurred later than 30 days post-surgery until the last follow-up visit).

Severe complications were defined as:

- Postoperative bleeding, with severe bleeding defined as the need for transfusion or hemodynamic instability secondary to bleeding;
- Thromboembolic events, either venous (deep venous thrombosis [DVT], pulmonary embolism [PE]), or arterial (valve thrombosis, myocardial infarction), as well as cerebrovascular events (transient ischemic attack [TIA] or stroke, limb ischemia or other arterial thrombosis) were diagnosed clinically and corroborated by an appropriate imaging study (Doppler ultrasound, computed tomography, etc.);
- Infections including endocarditis, bacteremia or other infection necessitating systemic antibiotic treatment;
- Post-pericardiotomy syndrome (PPS);
- Requirement for further operation;
- Death.

The study was approved by the institutional review board at each center (0409-21-TLV and 2610-15-SMC) and did not require the informed consent of the participating subjects since it was a retrospective analysis.

#### Statistical Analysis

Categorical variables are presented as total numbers and percentages, and continuous variables are presented as mean ± standard deviation (SD) or median (range). Univariate analysis was conducted to detect associations between the variables and the primary outcomes of severe complications and mortality. Chi-square or Fisher exact tests were applied to categorical variables, and an independent T-test was applied to continuous variables. Due to the small sample size, we could not conduct an adjusted multivariate analysis. The statistical analysis was performed with IBM SPSS Statistics software for Windows, version 27 (IBM, Armonk, NY, USA, 2020).

## 3. Results

We identified 26 patients who underwent cardiac valve surgery from 1992 to 2021. All patients but one, were classified as APS according to the Sapporo criteria [1], whereas one patient presented with high titer aPL, severe mitral regurgitation (MR) secondary to LSE but without prior APS thrombotic or obstetric-classifying manifestations. Twenty patients (76.9%) were female, the median age at surgery was 47.5 (range 18–71) years. Eleven patients (42.3%) sustained APS secondary to SLE. The median time from APS diagnosis to valve surgery was 17 years (range 0–40). Triple positive aPL was documented in 19 (79.2%) of the patients with available data (*n* = 24, data on LA was unavailable for 2 patients), of which, 22/24 (91.7%) had positive LA, 20 (76.9%) had moderate-to-high titers of aCL IgG antibodies and 18 (69.2%) had moderate-to-high titers of aB2GPI IgG antibodies. The APS manifested as arterial (*n* = 21, 80.8%), venous (*n* = 10, 38.5%), and/or obstetric (*n* = 6 out of 20 female subjects, 23.1%) (Table 1). 

The mean ± SD follow-up period following surgery was 62.69 ± 77.51 (range 0–347) months, for a total of 1630 patient months. 

### 3.1. Cardiac Pathologies in APS Patients Requiring Valve Surgery

The mitral valve was the most commonly affected valve with moderate-to-severe involvement (*n* = 15, 57.7%). Twelve of those patients had MR (80%) and three had mitral stenosis (MS) (20%). The second most commonly involved valve was the aortic valve (*n* = 9, 34.6%), with eight (88.9%) of those patients having aortic regurgitation (AR), one patient having concomitant severe aortic stenosis (AS), and one having isolated AS. The tricuspid valve was significantly involved in six patients (23.1%), four with tricuspid regurgitation (TR) and two with tricuspid stenosis. None of the patients had pulmonic valve involvement. Multiple valves were involved in eight patients (30.7%), including one with three affected valves (severe AR and AS, moderate TR and mild MR), four with concomitant mitral and tricuspid valve involvement and three with mitral and aortic valve involvement. 

Echocardiographic findings compatible with LSE were present in 15 (57.7%) of the study patients. 

The median NYHA functional class was 2 (range 1–4), including 10 patients (38.5%) who had a NYHA class of 3–4. Notably, 23 patients were assessed for the probability of PHTN by echocardiography before surgery of whom 11 (47.8%) were estimated to have a high probability of pulmonary arterial pressure.

Coronary anatomy was documented by coronary angiography prior to surgery in 22 patients, of whom 7 (31.8%) had abnormal coronary arteries.

### 3.2. Heart Valve Surgery Characteristics

Only two heart valve surgeries were performed between 1990 to 2000, three between 2000–2010 and 21 since 2010 (13 of them after 2015).

Most surgeries involved valve replacement (*n* = 24, 92.3%). Valve replacement was performed by means of a mechanical valve in 16/24 (66.7%) cases and a biologic valve in 8/24 (33.3%) cases. One patient underwent coronary artery bypass grafting (CABG) during MV replacement surgery. The repair of an additional valve was required in 5 of the 24 valve replacements. Two patients underwent primary valve repairs, one of the mitral valve, and the other of the tricuspid valve for a total of seven valve repairs (26.9%).

### 3.3. Medical Treatment before and after Valve Surgery

Chronic medical treatment before surgery included aspirin in 12 (46.2%) patients, VKA in 10 (38.5%) and LMWH in 9 (34.6%) (Table 2). VKA was prescribed postoperatively in 20 (76.9%) patients. The administered immunomodulatory medications are shown in Table 3.

### 3.4. Primary Outcomes: Severe Complications and Mortality

#### 3.4.1. Severe Complications

Fourteen (53.8%) patients sustained severe complications, of whom four patients died for a total mortality rate of 15.4%.

Early (up to 30 days post-surgery) severe complications were recorded for eight (30.8%) patients, including two cases of post-pericardiotomy syndrome, one valve infection, one valve thrombosis, one stroke and one major bleeding, as well as two early deaths (7.7%).

Late (more than 30 days post-surgery) follow-up data were available for 23 patients (two had died early and one had no available post-surgical data). Ten of them (43.5%) had a severe complication: two died (8.7%), two sustained infectious complications (*Escherichia coli* bacteremia, and bacterial [*Hemophilus influenza*] endocarditis), two had newly developed LSE (one of them with valve thrombosis), another one had valve thrombosis with hemorrhagic stroke), one had major bleeding, one had an ischemic stroke, and one developed chronic pericarditis. Three patients required a redo-valve replacement four years (LSE and valve thrombosis), two years (valve thrombosis and hemorrhagic stroke), and six months (bacterial endocarditis) after the first surgery.

Reassuringly, 12 of the 23 patients with more than 30 days of follow-up (52.2%) were complication-free during long-term follow-up, despite the fact that three of them had sustained an early complication.

#### 3.4.2. Mortality

Analysis of the early mortality cases revealed that one was an SLE female in her fifth decade who presented with lupus nephritis, diffuse alveolar hemorrhage (DAH), and LSE. Her course was complicated by antibiotic-resistant multi-organism (carbapenem resistant *Klebsiella Pneumonia*, methicillin-resistant *Staphylococcus Aureus* and *Acinetobacter*) septic shock leading to her death. The other early death occurred in a female patient with SLE who was in her sixth decade and who died on postoperative day 14 due to catastrophic APS with an ischemic limb requiring amputation, mesenteric ischemia, and splenic and kidney infarctions. She was treated with pulse methylprednisolone and plasma exchange soon after surgery but did not survive.

The two late deaths occurred more than 30 days after surgery, and they included a male patient with primary APS who was on chronic dialysis and who died four years after MV replacement. He had sustained recurrent episodes of DAH and died due to *Klebsiella* line-sepsis when in his late forties. That case may not be directly attributable to a valve surgery complication. The other late death occurred 1.5 years after MV replacement and tricuspid valve repair, due to endocarditis with severe leg infection in a female patient in her sixth decade who had SLE.

### 3.5. Variables Associated with Severe Complications

**Figure 1:**
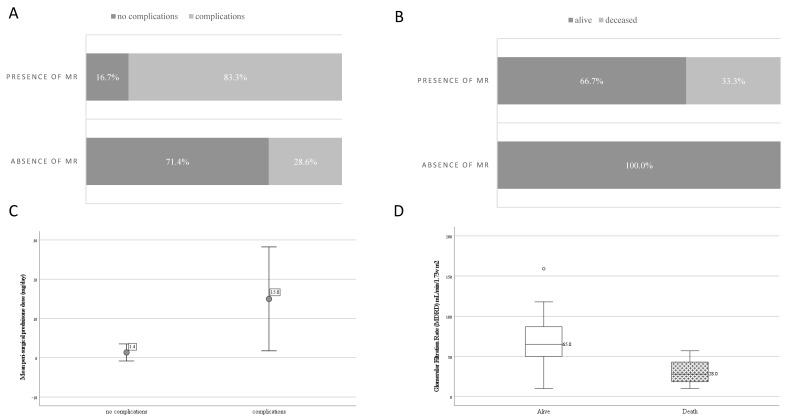
Variables significantly associated with total severe complications or mortality. Panel (**A**). Total severe complications associated with the presence of moderate-to-severe mitral regurgitation (MR), *p* = 0.008. Panel (**B**). Total mortality associated with presence of moderate-to-severe MR, *p* = 0.033. Panel (**C**). Mean ± standard deviation peri-surgical prednisone dose in mg/day in anti-phospholipid syndrome (APS) patients undergoing valve surgeries with and without severe complications, *p* = 0.046. Panel (**D**). Boxplot of glomerular filtration rate (GFR) (MDRD equation) in mL/min according to mortality of APS patients after valve surgery, *p* = 0.038.

A comparison of patients who had any severe complication following valve surgery including death (*n* = 14) to patients without severe complications (*n* = 11) revealed that the presence of moderate-to-severe MR (OR 12.5 (1.85–84.442), *p* = 0.008), LSE (OR 7.333 (1.272–42.294), *p* = 0.045) low C3 before surgery (OR 6.667 (1.047–42.431), *p* = 0.05) and a higher peri-surgical prednisone dose (mean ± SD 15 ± 21.89 vs. 1.36 ± 3.23 mg/day, *p* = 0.046) were associated with severe complications. Lack of chronic VKA treatment (OR for VKA 0.195 (0.035–1.084), *p* = 0.11), chronic LMWH treatment (OR 5.0 (0.79–31.627), *p* = 0.11) and low C4 (OR 8.25 (0.823–82.665), *p* = 0.08) were marginally associated with severe complications. Sex, age, APS duration, SLE diagnosis, and the probability of PHTN or type of prosthetic valve were not associated with complications (Figure 1 and Table 4).

### 3.6. Variables Associated with Mortality

The comparison of patients who died after valve surgery (*n* = 4) to those who survived (*n* = 22) demonstrated that the presence of moderate-to-severe MR (all of the deceased patients had MR, *p* = 0.033) and a lower glomerular filtration rate (GFR) (MDRD) (mean ± SD 30.75 ± 19.47 vs. 70.68 ± 34.44 mL/min, respectively, *p* = 0.038) were significantly associated with mortality. Interestingly, all of the patients who died had arterial APS manifestations, LSE and valve replacement with a mechanic prosthetic valve, although these variables did not reach a level of significance (Table 4). Sex, age and APS duration were not associated with mortality (Figure 1 and Table 5).

## 4. Discussion

The findings of this retrospective cohort study of 26 APS patients from two tertiary medical centers in Israel who underwent cardiac valve surgery in the last three decades emphasize the high morbidity and mortality rate in these patients compared with the general population.

The pathophysiology of valvular damage in APS is considered to involve endothelial damage by aPL deposition, specifically aCL, and ensuing thrombin formation, with initiation of an inflammatory process. This process is manifested pathologically by fibrin deposits, vascular proliferation, fibrosis and calcification, scant inflammatory infiltrates, ribbon-like aCL-IgG deposition and granular complement deposition [9,26,27,28].

MR is the most common valvular pathology observed in the general population undergoing valve surgery, as is in the APS patient population [3,4,8,12,13,14]. MV repair is the preferred procedure over replacement [29], the patients are older (mean 64.4–64.7 years) and they are more often male (45.8–59.1%) [30] as opposed to our cohort of APS patients undergoing valve surgery. Zhou et al. reported the mortality rate associated with MV replacement in the general population to be 7.8% for all procedures and 5% for isolated MV replacement, while MV repair entailed a lower mortality rate of 4.2% for all procedures and 1.7% for isolated MV repair [30]. The majority of our patients underwent MV surgery that mostly comprised MV replacement. Interestingly, all of our patients who died had undergone MV replacement.

The early mortality rate in our cohort was similar to the early mortality rate associated with MV replacement in the general population. It is encouraging to observe that APS patients may have comparable early outcomes, although better outcomes could be expected in this younger group of patients.

The literature regarding cardiac valve surgery in APS patients and particularly morbidity and mortality following this procedure is scarce and includes only a limited number of case reports and case series. A previous Israeli single-center case series [21] reported 10 patients who underwent valve surgery between 1989–2002. Those authors observed higher mortality and higher complication rates compared to those of our cohort with a total mortality rate of 40%, an early mortality rate of 20%, and a total complication rate of 80%, the latter including pulmonary hemorrhage, TIA, splenic infarction, and stroke. Despite early complications, 40% of the patients had an uneventful long-term follow-up.

The morbidity and mortality rates in more recent case series were similar to those of our study. In the largest case series published to date involving a multicenter study from England, Mexico and Spain, Erdozain et al. [16] reported 32 patients who underwent 33 valve replacements from 1981 to 2008. Four of those patients died (12.5%) and the total complication rate was 50%, including infections (sepsis, infectious prosthetic valve endocarditis), cardiac (cardiogenic shock, atrioventricular block, heart failure), hemorrhagic and thrombotic (arterial thromboembolism, TIA, valvular thrombosis, etc.) events. The year of surgery was associated with mortality, with none of the deaths occurring in the most recent period (2001–2008). The possible association with the year of surgery may explain the higher complication and mortality rates in the above-cited Israeli series which reported surgery outcomes before 2002. This association could not be evaluated in the present study since the majority of surgeries in our cohort were performed after 2010.

In another case series, Arif et al. [31] reported 15 patients, most of them with secondary APS, who underwent cardiac valve surgery with a mortality rate of 20% (none in the early postoperative period). The total complication rate was 60%.

Lastly, Colli et al. [22] reported nine patients from 1998–2007 who had a 33.3% total mortality rate and a 66.7% complication rate, with four (44.4%) of those patients having had an uneventful long-term follow-up. Complications included thromboembolic and hemorrhagic events.

Smaller case series, some including other cardiovascular operations [32,33,34], reported even higher rates of severe complications (ranging from 66.7% to 84.2%) and mortality (from 33.3% to 63.6%). Gorki et al. [14] performed a meta-analysis of 57 cases of valve surgery in patients with APS from 1988–2007, including the Israeli series reported by Berkun et al. [21]. The early mortality rate in that case-report meta-analysis was 7%, and the late mortality rate was 12% (mean follow-up of three years), while 42% of the patients had an uneventful short- and long-term follow-up.

Surgical intervention in APS patients in general carries thrombotic and hemorrhagic complication risks. Other studies have dealt with a variety of surgeries in APS patients [19,33,34]. One retrospective analysis of 48 elective surgeries in APS patients included nine (19%) cardiovascular surgeries, of which two were valve replacements [19]. All 48 patients were treated with VKA before surgery and were bridged with LMWH or unfractionated heparin. As was observed in our current study, the APS patients sustained thrombotic events (three patients) hemorrhagic complications (six patients, one with both PE and severe bleeding) and infectious complications (six patients). In accordance with our results, that study did not find any association between surgical complications and age, the coexistence of SLE, or the specific serological profile, namely aPL isotype or number of positive aPL.

Taken together, the vast majority appear to report a relatively high rate of complications following surgeries in general, and heart valve surgery in particular, among APS patients in comparison to patients who undergo valve surgery for other causes. The increased rates of morbidity and mortality in this young population are mostly due to thrombotic and or hemorrhagic complications inherent to the nature of APS and its treatment as well as to an increased risk of infection due to immunosuppressive medications.

Importantly, we detected several factors associated with worse outcomes. Moderate-to-severe MR was associated with severe complications and mortality, similar to reports in the general population where MV replacement carries higher complication and mortality rates compared to other valve surgeries [30,35]. LSE valvular lesions were also associated with severe complications. Interestingly, all of the patients in our cohort who had died had LSE.

We also report additional factors associated with complications in APS patients undergoing heart valve surgery, including low complement levels, high dose prednisone, and low GFR. To the best of our knowledge, this is the first report of an association between low complement levels and severe complications in APS patients undergoing cardiac valve surgery. Indeed, complement activation has been reported to play an important role in the pathophysiology of APS [36,37]. Low complement levels have been associated with obstetric complications [38], and may serve as a marker of high-risk APS patients as well.

The medical treatment of APS patients with the valvular disease is debated and usually consists of anticoagulation in symptomatic patients and anti-aggregation in asymptomatic patients [5]. Accordingly, a larger proportion of patients in our cohort received VKA after than before the surgical procedure. Regarding immunosuppressive treatments, the data is less clear, with some indicating the progression of vegetation to fibrosis and some suggesting a milder course [5,18]. In our cohort, only a small fraction of the patients received immunosuppression, usually in order to address other severe manifestations of their disease. We found that high doses of prednisone were associated with worse outcomes. High prednisone doses peri-surgically may be a marker of high-risk patients since high-dose prednisone is mostly prescribed when managing life- or organ-threatening manifestations of the disease, which may contribute to postoperative complications. In addition, the use of glucocorticoids may predispose patients to infectious and hemorrhagic complications.

Lastly, low GFR was associated with a higher risk of death. A similar association between a low GFR and risk of death was shown in the general population requiring cardiac surgery [39]. Specifically, this association may even be more significant in the APS patient population due to the challenge of achieving adequate anticoagulation post-surgery in patients with a low GFR. 

Our retrospective report has several limitations. The small number of patients limits the statistical power, results in wide confidence intervals, inability to do sensitivity analyses and restriction of the ability to reliably assess risk factors for morbidity and mortality. The retrospective nature, the limited number of patients, and the lack of randomization can contribute to selection bias. In addition, APS is a complex disease with a myriad of manifestations that can introduce confounding bias, which we could not properly address. The strengths of this study include a cohort from two large referral centers, and a relatively large number of cases compared to other series. Case series are less prone to publication bias than case reports and, as such, our series contributes to extending the knowledge regarding the prognosis of cardiac valve surgery in APS patients. Despite this being a case series, we were able to identify meaningful clinical factors associated with a worse outcome. 

## 5. Conclusions

Herein, we report that heart valve surgery in patients with APS is linked with perioperative and late morbidity as well as mortality, which may be higher than expected for age compared to the general population. Surgery in APS patients carries higher risks arising from their disease (thromboembolism) and the treatment they receive (bleeding and infections), and these risks should be screened for and addressed during preparation for surgery and follow-up after the procedure. We have identified possible predictors of poor outcomes among APS patients, namely moderate-to-severe MR, LSE, low complement levels, higher glucocorticoid doses peri-operatively, and low GFR.

Larger studies and registries are needed to define risk factors for severe valvular involvement and those for adverse outcomes following valve surgery, as well as delineating the best surgical approach and peri-operative medical management.

## Figures and Tables

**Table 1:** Demographic and clinical characteristics of APS patients undergoing heart valve surgery in 1992–2021.

	APS Patients (*n* = 26)
Female subjects *n* (%)	20 (76.9)
Age, years mean ± SD	45.6 ± 13.4
SLE, *n* (%)	11 (42.3)
APS disease duration, years mean ± SD	16.1 ± 12.8
**Immunologic laboratory values**	
aCL-IgM positive *n* (%)	5 (19.2)
aCL-IgG positive *n* (%)	20 (76.9)
aB2GPI-IgM positive *n* (%)	5 (19.2)
aB2GPI-IgG positive *n* (%)	18 (69.2)
LA positive (*n* = 24) *n* (%)	22 (91.7)
Triple positive (*n* = 24) *n* (%)	19 (79.2)
Direct Coombs positive *n* (%)	5/24 (20.8)
C3 titer mean ± SD	87.3 ± 19.3
Low C3 < 85 *n* (%)	10 (38.5)
C4 titer mean ± SD	16.6 ± 7.1
Low C4 < 15 *n* (%)	7 (26.9)
**APS manifestations** *n* (%)	
Any venous thromboembolism	10 (38.5)
Deep vein thrombosis	10 (100)
Pulmonary embolism	5 (50)
Arterial thrombosis	22 (84.6)
Stroke	12 (46.1)
Transient ischemic attack	4 (15.4)
Myocardial infarction	3 (11.5)
Peripheral arterial thrombosis (limb, spleen, etc.)	11 (42.3)
Obstetric manifestations *n* = 20	6 (30)

APS, antiphospholipid syndrome; *n*, number; SD, standard deviation; SLE, systemic lupus erythematosus; aCL, anticardiolipin; Ig, immunoglobulin; aB2GPI, anti-beta-2 glycoprotein I; LA, lupus anticoagulant.

**Table 2:** APS medical treatment before, perioperatively and following heart valve surgery.

Treatment	Chronic Medical Therapy before Surgery	Medical Therapy after Surgery
Aspirin	12 (46.2)	11 (42.3)
VKA	10 (38.5)	20 (76.9)
LMWH	9 (34.6)	4 (15.4)
DOAC (rivaroxaban)	1 (3.8)	0
Fondaparinux	2 (7.7)	1 (3.8)
Heparin	0	1 (3.8)

APS, antiphospholipid syndrome; VKA, vitamin K antagonists; LMWH, low molecular weight heparin; DOAC, direct oral anticoagulation.

**Table 3:** APS immunomodulatory and immunosuppressive treatments before and in the perioperative period of heart valve surgery.

Immunotherapy	Before Hospitalization for Surgery Number/Total with Available Data (%)	Peri-Surgical Period, Number/Total with Available Data (%)
Pulse GC	1/25 (4)	3/24 (12.5)
Hydroxychloroquine	8/25 (32)	7/24 (29.2)
Azathioprine	5/25 (20)	4/24 (16.7)
Rituximab	2/25 (8)	2/24 (8.3)
Cyclophosphamide	1/25 (8)	1/24 (4.2)
Plasma exchange	1/25 (4)	3/24 (12.5)
IVIg	2/25 (8)	2/24 (8.3)
Mycophenolate mofetil	0	0
Belimumab	1/25 (4)	0

APS, antiphospholipid syndrome; GC, glucocorticoids; IVIg, intravenous immunoglobulins.

**Table 4:** Variables associated with severe complications in APS patients undergoing heart valve surgery.

Variable *n*(%)	Total Severe Complications (*n* = 14)	Uneventful Follow-Up (*n* = 11)	OR (95%CI)	*p*-Value
Female subjects	11 (55)	9 (45)	1.222 (0.197–7.594)	>0.99
Age at surgery (mean ± SD)	47.86 ± 13.92	43 ± 12.842		0.367
APS with SLE	6 (54.54)	5 (45.45)	1.05 (0.22–5.003)	>0.99
Positive LA **	12 (54.5)	10 (45.5)	1.2 (0.066–21.723)	>0.99
Triple positive aPL **	10 (52.6)	9 (47.4)	0.741 (0.1–5.49)	>0.99
Low C3	8 (80)	2 (20)	6.667 (1.047–42.431)	**0.051**
Low C4	6 (85.7)	1 (14.3)	8.25 (0.823–82.665)	0.081
APS duration	12.11 ± 13.36	20.79 ± 10.83		0.084
Platelet count, 10 × 10^3^/µL	127.07 ± 44.17	118.91 ± 60.73 *		0.701
Hemoglobin, g/dL	10.87 ± 1.53	11.81 ± 1.41 *		0.127
Creatinine mg/dL	1.88 ± 1.78	1.12 ± 0.4 ***		0.146
eGFR ml/min/1.73 m (MDRD)	57.14 ± 34.64	74 ± 36.39 ***		0.277
**APS manifestations**				
VTE	4 (40)	6 (60)	0.333 (0.063–1.752)	0.241
CVA/TIA	8 (61.5)	5 (38.5)	1.6 (0.326–7.848)	0.695
Arterial APS	12 (57.1)	9 (42.9)	1.333 (0.157–11.356)	>0.99
**Cardiac pathology**				
LSE	11 (73.3)	4 (26.7)	7.333 (1.272–42.294)	**0.045**
Moderate-to-severe MR	10 (83.2)	2 (16.7)	12.5 (1.85–84.442)	**0.008**
Moderate-to-severe AR	3 (37.5)	5 (62.5)	0.382 (0.069–2.125)	0.401
Moderate-to-severe TR	3 (75)	1 (25)	3 (0.269–33.487)	0.598
Intermediate-high probability of PHTN	7 (36.4)	4 (63.6)	1.75 (0.329–9.298)	0.680
NYHA class	2.21 ± 0.96	2.25 ± 0.866		0.923
Surgery year > 2015	8 (61.5)	5 (38.5)	1.867 (0.392–8.894)	0.695
**Prosthetic valve**				
Only repair	1 (50)	1 (50)	NA	0.348
Mechanic	7 (43.8)	9 (56.3)		
Biologic	6 (75)	2 (25)		
CABG	1 (100)	0 (0)		>0.99
Post-op redo	3 (100)	0		0.225
**Medical treatment**				
**Background therapy**				
VKA	3 (30)	7 (70)	0.195 (0.035–1.084)	0.105
Aspirin	7 (58.3)	5 (41.7)	1.4 (0.296–6.622)	0.713
LMWH	7 (77.8)	2 (22.2)	5.0 (0.79–31.627)	0.11
Prednisone dose mg/day	7.14 ± 15.78	1.36 ± 2.34 *		0.243
Peri-surgical prednisone dose mg/day	15 ± 21.89 *	1.36 ± 3.23 *		**0.046**
**Post-surgical therapy**				
VKA	10 (50)	10 (50)	0.5 (0.074–3.378)	0.652
Aspirin	6 (54.5)	5 (45.5)	1.05 (0.22–5.003)	>0.99
LMWH	3 (75)	1 (25)	3 (0.269–33.487)	0.598
Prednisone dose mg/day	14.62 ± 23.32 *	1.36 ± 3.23 *		0.065

* data are missing for 1 participant ** data are missing for 2 participants *** data are missing for 3 participants. Continuous variables are presented as means ± standard deviations, and categorical variables are presented as numbers (percent). APS, antiphospholipid syndrome; *n*, number; OR, odds ratio; CI, confidence interval; SLE, systemic lupus erythematosus; LA, lupus anticoagulant; aPL, antiphospholipid antibodies; C3/C4, complement 3/4; eGFR, estimated glomerular filtration rate; MDRD, modification of diet in renal disease formula; VTE, venous thromboembolism; CVA/TIA, cerebrovascular accident/transient ischemic attack; LSE, Libman-Sacks endocarditis; MR, mitral regurgitation; AR, aortic regurgitation; TR, tricuspid regurgitation; PHTN, pulmonary hypertension; NYHA, New York Heart Association; CABG, coronary artery bypass grafting; VKA, vitamin K antagonists; LMWH, low molecular weight heparin.

**Table 5:** Variables associated with mortality in APS patients undergoing heart valve surgery.

Variable *n*(%)	Total Mortality *n* = 4	No Mortality *n* = 22	OR (95%CI)	*p*-Value
Female subjects	2 (10)	18 (90)	0.222 (0.024–2.086)	0.218
Age at surgery	48.25 ± 6.95	45.14 ± 14.32		0.678
APS with SLE	3 (27.3)	8 (72.7)	5.25 (0.465–59.286)	0.279
Positive LA **	2 (9.1)	20 (90.9)	0.1 (0.004–2.287)	0.239
Triple positive aPL **	1 (5.3)	18 (94.7)	0.083 (0.006–1.232)	0.099
Low C3	2 (20)	8 (80)	1.75 (0.205–14.931)	0.625
Low C4	1 (14.3)	6 (85.7)	0.889 (0.077–10.3)	>0.99
Platelet count, 10^3^/µL	121.5 ± 55.82	123.86 ± 51.62 *		0.935
Hemoglobin, g/dL	10.75 ± 1.7	11.38 ± 1.51 *		0.46
Creatinine mg/dL	2.65 ± 1.55	1.36 ± 1.35		0.105
eGFR ml/min/1.73 m (MDRD)	30.75 ± 19.47	70.68 ± 34.44		**0.038**
APS duration	14.75 ± 17.35	16.36 ± 12.32		0.822
**APS manifestations**				
VTE	3 (30)	7 (70)	6.0 (0.524–68.719)	0.267
CVA/TIA	3 (23.1)	10 (76.9)	3.3 (0.294–37.103)	0.593
Arterial APS	4 (19)	17 (81)	NA	>0.99
**Cardiac pathology**				
LSE	4 (26.7)	11 (73.3)	NA	0.113
Moderate-to-severe MR	4 (33.3)	8 (66.7)	NA	**0.033**
Moderate-to-severe AR	1 (12.5)	7 (87.5)	0.714 (0.063–8.15)	>0.99
Moderate-to-severe TR	2 (50)	2 (50)	10.0 (0.871–114.746)	0.099
Intermediate-high probability of PHTN	2 (18.2)	9 (81.8)	1.11 (0.129–9.605)	>0.99
NYHA class	2.25 ± 1.5	2.23 ± 0.81		0.978
Surgery year > 2015	1 (7.7)	12 (92.3)	0.278 (0.025–3.104)	0.593
**Prosthetic valve**				
Only repair	0 (0)	2 (100)	NA	0.228
Mechanic	4 (25)	12 (75)		
Biologic	0 (0)	8 (100)		
CABG	1 (100)	0 (0)	NA	0.154
**Medical treatment**				
**Background therapy**				
VKA	1 (10)	9 (90)	0.481 (0.043–5.401)	>0.99
Aspirin	3 (25)	9 (75)	4.33 (0.386–48.61)	0.306
LMWH	3 (33.3)	6 (66.7)	8 (0.69–92.703)	0.104
Prednisone dose mg/day	18.75 ± 27.8	1.9 ± 3.35 *		0.312
Peri-surgical prednisone dose mg/day	20 ± 27.39	6.5 ± 14.7 **		0.161
**Post-surgical therapy**				
VKA	3 (15)	17 (85)	0.882 (0.074–10.464)	>0.99
Aspirin	2 (18.2)	9 (81.8)	1.444 (0.171–12.232)	>0.99
LMWH	1 (25)	3 (75)	2.111 (0.162–27.582)	0.511
Postsurgical prednisone dose mg/day	32.5 ± 32.02	3.75 ± 9.85 **		0.17

* data are missing for 1 participant ** data are missing for 2 participants. Continues variables are presented as means ± standard deviations, categorical variables are presented as numbers (precent). APS, antiphospholipid syndrome; *n*, number; OR, odds ratio; CI, confidence interval; SLE, systemic lupus erythematosus; LA, lupus anticoagulant; aPL, antiphospholipid antibodies; C3/C4, complement 3/4; eGFR, estimated glomerular filtration rate; MDRD, modification of diet in renal disease formula; VTE, venous thromboembolism; CVA/TIA, cerebrovascular accident/transient ischemic attack; LSE, Libman-Sacks endocarditis; MR, mitral regurgitation; AR, aortic regurgitation; TR, tricuspid regurgitation; PHTN, pulmonary hypertension; NYHA, New York Heart Association; CABG, coronary artery bypass grafting; VKA, vitamin K antagonists; LMWH, low molecular weight heparin.

## Data Availability

Research data is available from the corresponding author upon reasonable request.
